# Establishing a New Ambulatory Care Practice Site as a Pharmacy Practice Faculty

**DOI:** 10.3390/pharmacy6040111

**Published:** 2018-10-11

**Authors:** Vasudha Gupta, Evan Williams

**Affiliations:** College of Pharmacy, Roseman University of Health Sciences, 11 Sunset Way, Henderson, NV 89014, USA; ewilliams1@roseman.edu

**Keywords:** ambulatory care, pharmacy practice faculty, pharmacy learners, clinical practice, pharmacist services

## Abstract

There is an imminent need to identify and develop new ambulatory care practice sites with the increase in the number of colleges of pharmacy across the nation. This manuscript provides recommendations to help clinical faculty determine whether a potential pharmacy practice site will be able to provide adequate resources and support to establish a successful practice. This may be challenging to pharmacy practice faculty in settings where clinical pharmacy services have never been utilized. Topics include the pre-work needed prior to approaching a new practice site, assessing the need for physical requirements, meeting key personnel, marketing clinical skills and services, implementing, and evaluating practice site. Preparation includes having a clear vision of the pharmacist services, ensuring that stakeholders have an understanding of the pharmacy services inquiring the site support and resources for the pharmacist, and regularly communicating.

## 1. Purpose

The Accreditation Council for Pharmacy Education (ACPE) Standards 2016 requires all colleges of pharmacy across the United States to provide ambulatory care practice experiences to pharmacy students as a requirement for graduation [1]. With the increase in the number of colleges of pharmacy across the nation, there is an imminent need to identify and develop new ambulatory care practice sites [2,3]. This provides unique challenges for pharmacy practice faculty, as well as colleges of pharmacy, to establish a rewarding clinical practice while also providing opportunities for learners, including pharmacy students and residents. Additionally, faculty may be working with providers who may be unfamiliar with the clinical services ambulatory care pharmacists can provide [4]. Since many of these new programs are being established in areas where ambulatory care clinical pharmacy services are relatively novel, pharmacy practice faculty often find themselves describing the utility of an ambulatory care pharmacist. 

Previous publications have focused on the establishment of a practice site that will exhibit clear evidence of outcome improvement and create financial incentives for clinics to thrive and justify salary requirements [5,6,7,8,9,10]. Pharmacy practice faculty have a different priority when establishing new ambulatory care clinic sites. Thus, it is imperative that the pharmacy practice faculty and academic unit closely evaluate the practice site for suitability towards their purpose. This purpose includes a collaborative learning environment to foster development of learners and building a practice site in his or her area of expertise. Literature is lacking for faculty hopeful in establishing a new practice site that will fulfill their goals and objectives. Based on experience establishing new ambulatory care practice at sites which have not previously had a pharmacy practice faculty, the authors have identified various factors to consider in order to create a successful practice model. These experiences include creating disease-state specific clinics, initiating interprofessional patient health programs, improving meaningful use and quality metrics as part of accountable care organizations, and coordinating activities at multiple clinics within an organization. The authors have also had experience with establishing practice initiatives that did not thrive to their full potential, allowing for reflection on the key issues that lead to unsuccessful implementation and identification of strategies to improve future forays.

Evaluation of the practice site is a multifaceted process and the importance of each section below needs to be weighed determine if the site will fit the needs of the pharmacist and the college of pharmacy. As a result, a formulaic approach is unlikely to be successful as each situation will require a nuanced understanding of various factors. The recommendations below are not in a sequential order and may need to be adapted to circumstances and preliminary conversations with the practice site.

## 2. Summary

### 2.1. Preparation

Prior to a meeting with individuals at the practice site, the first discussion that will occur will be between the pharmacy practice faculty and the college leadership, such as the dean of the college of pharmacy, clinical department chair or experiential director. This conversation will be focused on various responsibilities of the pharmacy practice faculty, related to expectations for both didactic and experiential teaching, service to the college and the profession, as well as scholarly activities. It’s critical for the faculty and the college leadership to establish expectations related to how much time will be devoted to each of the faculty’s roles, and specifically the time devoted to the practice site. Additional consideration should be given if the faculty position is split funded, with the clinic or health system providing some salary offset, either directly to the pharmacist or though remuneration to the school. In these situations, the clinic may have higher expectations regarding how much time the faculty member should be providing services at the site. Thus, clear delineation of roles and responsibilities is crucial during initial conversations between the college and the practice site leadership. As successful implementation of a new practice site may take up to 6–12 months, additional time may be devoted to the practice site during that period, with limited focus on other faculty responsibilities. The time commitment of the pharmacy practice faculty to the practice site will need to be discussed again in collaboration with the clinic leadership to establish clear expectations.

A practice protocol may help pharmacy practice faculty interested in pursuing a new practice site. The protocol can describe proposed clinical services and how the service may fit patients’ needs, outlines key components of the practice flow, and includes the goals and the vision for the pharmacy services. The goals and vision may include conducting comprehensive medication reviews, identifying and solving complex drug-related problems, ensuring evidence-based and cost-effective therapies, and educating patients about appropriate medication use. Faculty also need to identify which disease states they would like to address, the types of collaborative drug therapy management that would be offered, and the scope of practice desired. Although it’s essential to have a vision for services based upon the faculty’s practice interest, it’s important to keep in mind that the actual services provided at the site may be modified based upon a needs assessment of the patients seen at the practice site. However, the services may be extended after a period of time to also include some of the faculty’s practice interest.

The description should include information such as hours of operation of the clinical services, how patients will be referred, what will be accomplished during the appointment with the patient, how interactions with patients will be documented, changes to therapy communicated to providers, what follow-up will look like, and how providers will be able to contact the pharmacist. Beyond the clinical components of practice, faculty also need to consider how the environment will foster teaching and learning, including opportunities for interprofessional education. Writing these components into a practice protocol will help define the pharmacist’s role in patient care, but may need to be revised after discussions with key clinic personnel. 

Faculty should consider gathering some evidence demonstrating that pharmacy services can help improve patient care in various practice settings as part of the preparation [11,12,13,14]. The pharmacist should also have an updated curriculum vitae (CV) that includes his or her training and experiences in implementing pharmacy services to help key clinic personnel to gain an understanding of role of the pharmacist.

Laws regarding the scope of practice and collaborative drug therapy management (CDTM) play a large role in delineating what is achievable in a new practice. This may be especially important in areas where ambulatory care clinical pharmacy services have not been fully established and widely adopted. Enhancing the scope of pharmacy practice may be difficult as it requires buy-in from several different stakeholders. While there are collaborative practice laws in nearly every American jurisdiction, the level of practicality written into these regulations varies widely across the nation and dictates limitations to the practice culture [15]. These laws must be considered in order to develop the clinical practice site. Consider preparing a collaborative practice agreement (CPA) or modify one that has been previously created taking state laws into consideration [16]. This may serve useful during conversations with the medical director to help develop a mutual understanding of the pharmacist’s scope of practice.

One barrier encountered involves unfamiliarity with the role of the pharmacy practice faculty in an ambulatory care practice. Resolution of this issue may require education about the pharmacist’s role in direct patient care prior to discussions regarding the model of practice at the clinic. It’s important to emphasize that the pharmacist’s role is to augment the productivity of the clinic and optimize patient outcomes in collaboration with other healthcare providers (HCPs) and does not replace the other providers’ patient encounters [3,4]. The Pharmacist Patient Care Process model (PPCP) has been created to assist with succinctly explaining the role of a pharmacist in patient care, and may prove useful for discussions with providers and other clinic staff [17]. 

Other issues that may arise and limit the full implementation of the original practice vision may be the patient population, reimbursement constraints, or access to health resources. Careful consideration of potential barriers must be taken and the original practice protocol revised as necessary to create a plan that can be implemented within the required boundaries.

### 2.2. Physical Requirements

Consideration of physical requirements including space, access, and equipment are important to successfully establish pharmacy services. The discussion regarding these essentials will likely need to occur in collaboration with college and clinic leadership as the finances related to any physical space modifications, electronic health record (EHR) access, supplies and equipment for the faculty as well as the students will need to be delineated.

#### 2.2.1. Space

Many clinic managers may be unfamiliar with the role of an ambulatory care pharmacist or have little insight as to the physical space and access requirements that a pharmacy practice faculty might need. Request adequate space for the number of patients that will be seen and the number of learners who will be on rotation at one time. There will be a need for an exam room or private space if direct patient care is offered. In the authors’ experiences, two exam rooms are optimal if patients are scheduled back-to-back (every 30 or 45 min), as this allows for the rooming of one patient while the pharmacist is seeing another patient, improving efficiency. 

Another key consideration for a successful practice is office space. Consideration regarding where documentation of encounters will be completed and where learners will look up information and prepare to interview patients is important. Determine the need for desks, chairs, work terminals, and storage space for educational materials. Having a dedicated workstation for the pharmacist and one workstation per learner is optimal. The pharmacist should also consider the space needed to have discussions with learners about work related to patient care or other assignments required for the rotation. Having access to a conference room, if available, provides adequate space to better facilitate topic discussions, and journal article presentations. 

#### 2.2.2. Access and Equipment

Electronic health record (EHR) access is key to any successful ambulatory care practice, and is becoming more prevalent even in community [18,19]. The clinic site must ensure that, as a HCP, the pharmacist will have access to the full medical record, as well as the ability to document in the EHR. The ability to modify medication orders will vary between locations based on local collaborative practice laws and practice culture. The pharmacist should advocate to practice at the top of his or her license and promote themselves as an integral part of the healthcare team. It may be appropriate for the pharmacist to initially attain limited authority to establish rapport with the providers until providers are comfortable with the pharmacist’s clinical decision making.

The pharmacist should consider the resources that are available for scheduling and seeing patients in addition to EHR access and permissions. Considerations may include equipment needed for practice. These resources can include a blood pressure machine or point of care testing devices, availability of a medical assistant to help with scheduling and taking vitals for patients, and a phone for contacting and following-up with patients. 

### 2.3. Meeting Key Personnel

#### 2.3.1. Finding a Niche

One of the first meetings that will likely occur is with the medical director and the manager of the clinic to assess their interest in clinical pharmacy services. The medical director will be an essential partner to advocate for the clinical pharmacy services to other providers as they oversee all the HCPs at the site. The clinic manager will be a key asset during day-to-day operations as the pharmacist is learning to practice in a new environment. Other key personnel, such as a compliance officer, other medical leaders, human resources, or chief financial officer, may also be present during one of the first meetings to provide additional perspectives.

Pharmacy practice faculty must inquire about the patient population seen at the clinic and the needs of the patients from the medical director and clinic manager. Knowing basic information such as patient age, literacy, ethnicity, and socioeconomic status will help to tailor services to meet patients’ needs. Ask the medical director about any current needs of the clinic or any gaps in care that the providers are hoping to improve. Identifying these will aid in matching the skill-set of the pharmacist with the needs of the site, creating a symbiotic relationship. 

Faculty should provide a draft of the practice protocol for overview, which details a description of proposed clinical services and outlines key components of the flow of the practice. This will allow the medical director and the clinic manager to have a sound understanding of the day-to-day operations and how the patients will benefit from the pharmacist consultations. Some processes will likely change depending on the needs assessed during the discussion as well as the limitations of the clinic. 

#### 2.3.2. Marketing Your Skills and Services to the Medical Director and Clinic Manager

The pharmacist should describe the importance of the program, and the vision of progression of services, including both short and long-term goals during the meeting. Goals could include expanding the scope of the disease states managed, initiating group patient education classes, or regularly planning a short presentation with providers at the clinic for continuing education. General goals of the service should include delivery of comprehensive, integrated, and patient-centered care to improve patient health and reduce costs. As the climate regarding medical service reimbursement shifts from fee-for-service towards improving quality metrics, the efforts of the pharmacist can be focused to ensure that compensation is maximized. Within certain value-based reimbursement initiatives, achieving population health goals, such as a certain percentage of cardiovascular disease (CVD) patients receiving an appropriately dosed statin, can increase the rate of reimbursement to the clinic. Myriad examples of pharmacist-led initiatives in quality improvement exist, and it is recommended that the faculty member become familiar with current quality-based reimbursement models prior to meeting with the medical director and clinic manager [11,13,14]. In the authors’ experience, improving just a few quality measures for the clinic often opens doors to a broader practice scope as the clinic management observes the positive impact the pharmacist has on patient care.

The faculty can consider discussing how pharmacists can help improve care by reducing medication errors, decreasing number of medications by discontinuing inappropriate medications, optimizing therapy to meet therapeutic goals, improving patient outcomes, improving patient satisfaction, increasing medication adherence, enhancing patient understanding, reducing patient and health-system costs, and decreasing hospitalizations [20,21,22,23,24,25,26,27,28]. The authors have found the Ambulatory Care Pharmacist’s Survival guide to be a useful resource which highlights successful pharmacy practice initiatives, along with networking through national organizations, including the American Association of Colleges of Pharmacy (AACP), the American College of Clinical Pharmacy (ACCP), and the American Society of Health-Systems Pharmacists (ASHP) [6]. These organizations, and others like them, hold national and international meetings in which presenters highlight their practice initiatives and provide a platform for networking and establishing mentoring relationships with other practice faculty. Seeking out these mentoring relationships is recommended and can provide an excellent resource for academic pharmacists establishing ambulatory care practice sites.

#### 2.3.3. Other Points of Discussion

Billing for pharmacy services may not be a requirement as a pharmacy practice faculty. However, inquiring about the billing structure may be important to consider if the site is amenable to billing patients for pharmacist services. Clinics participating in a split-funded faculty position may have a greater stake in ensuring revenue is generated from the pharmacy services. This revenue can aid the clinic in recuperating some of the cost of the pharmacist’s salary, or can be used to provide more resources for the pharmacist to improve patient care. Although a discussion regarding billing practices are beyond the scope of this manuscript, many resources are available to practicing pharmacists regarding billing for clinical services [9,29,30,31]. Knowing the most common types of insurances that will be encountered at the site will also help to determine what medications are normally covered by insurance versus those that are unlikely to be covered. 

The pharmacist should discuss the implementation of a collaborative practice agreement (CPA) in an effort to gain autonomy in decision making. As most providers may not be familiar with a CPA, providing a copy of one that was developed by the pharmacist or a sample copy that is available through resources may be helpful [6,7,20]. A collaborative practice may have a narrow or broad scope which will depend on state laws, as well as the comfort level of providers to allow a pharmacist to make clinical decisions regarding patient care. Discussion should focus on the benefits of a collaborative practice agreement [32,33,34,35]. In the authors’ experiences, this includes easing care burden of providers, avoiding provider workflow interruptions to inquire about a patient, and improving the timeliness of patient care. 

#### 2.3.4. Keeping Faculty Responsibilities in Mind

A pharmacy practice faculty is likely required to precept pharmacy students and perhaps residents at the practice site; thus, it is important to discuss this role with the medical director and clinic manager. Explain that the learners, especially pharmacy residents, will operate as an extension of the pharmacist and will be able to closely monitor patients seen at the clinic under supervision. Learners can help coordinate in-services for providers, as well as complete projects that will improve care for patients and help to improve clinic functions [36,37]. Assess the clinic for opportunities where learners would be able to learn about, from, and with other healthcare professional learners [38]. Discuss how interprofessional education can allow for effective collaboration and allow all those involved to have a better understanding of their respective roles and responsibilities, develop a mutual respect for interprofessional practice, improve communication, and function more cohesively as a team to improve patient care. This has been identified as an essential component in maintaining ACPE accreditation and improving patient care [1,39].

An additional pharmacy practice faculty responsibility will likely be an expectation of research in an area of expertise. Clinical practice aligns with clinical research and provides an environment where new ideas may develop based upon scenarios in patient care. Inquire about the feasibility of research at the practice site, including the ability to collect patient information, availability of resources to help with data collection, the internal or external institutional review board (IRB) requirements at the site. Identifying opportunities early will help the pharmacist initiate research endeavors and plan for known obstacles.

### 2.4. Implementation

#### 2.4.1. Recommendations for Starting Out

The pharmacist should request to attend provider meetings prior to starting services at the site even though he or he will have met with the medical director and the clinic manager. The pharmacist should provide an introduction, his or her integration and role within the healthcare team, and the services that can be provided. Additionally, determining areas where the HCPs feel that a pharmacist can provide value to the patients may help the HCPs buy into the new services, as it may directly help the patients.

The pharmacist should also consider attending staff meetings temporarily as they are getting started to become acquainted with the staff and help them understand the role of the pharmacist. Speaking with the staff may provide insight of the current systems and processes in place, and can also identify barriers and opportunities where the pharmacist might be able to help. 

#### 2.4.2. Evaluation

The pharmacist should regularly evaluate the success of current methods in achieving short and long-terms goals once clinical services have been established. Ask for feedback from either the medical director or HCPs at the site to determine if any changes need to be made to improve the integration of pharmacy services into the clinical workflow. Prior to starting services, it is important to identify some metrics which will define success for the ambulatory care pharmacist. These could include decrease in A1c, or blood pressure. Identify key personnel, such as an individual in the field of pharmacy informatics or information technology, who may be able to help navigate tracking data and designing reports with the desired outcomes. Learners can be helpful with tracking the clinical impact and sorting through data, which may be especially important for pharmacy practice faculty in their evaluations to achieve promotion and tenure. Showing the number of interventions the pharmacist and learners have made, care improvements that have occurred, and even estimating cost savings are all excellent ways to show that the contributions at the clinic are leading to positive outcomes for patients. This data can also be used for formalized research projects to be synergistic with the pharmacy practice faculty’s goals and aspirations towards promotion. 

## 3. Conclusions

Implementing a new clinical service at an ambulatory care site as a pharmacy practice faculty may be overwhelming but can also be exciting. Being aware of the appropriate information to gather prior to committing to a practice site is crucial in creating a valuable learning site. The pharmacist should ensure that an overview of the services that will be offered to the patients at the clinic has been provided, and inquire regarding the support and resources that can be expected from the site. Open communication can increase the likelihood of success at the practice site and provide a great opportunity to showcase the importance of interprofessional practice in ambulatory care clinics without previous pharmacist services. Successful implementation will be essential to the education of our future pharmacists. 


**Key Considerations**

Have a clear vision of the pharmacist services at the practice siteBe prepared prior to discussions with the medical director, other healthcare providers, and clinic manager, and anticipate questions that may ariseEnsure that the key stakeholders have a clear understanding of the services that the pharmacist will provide to the patients at the clinic, and inquire regarding the support and resources that the pharmacist can expect from the sitePrior to committing to a practice site, gather information regarding the feasibility of a successful clinic and seek mutually beneficial goals that will provide benefit and value to pharmacy students and residents, providers, patients, and to the pharmacy practice facultyRegular communication with the providers and staff will help identify barriers and help set up a thriving clinical practice siteRecognize that establishing a new practice is a difficult process, and it may take a considerable amount of time for the pharmacist to become fully integrated into the clinicDetermine needs versus wants and be prepared to negotiate to establish a successful learning environmentBe flexible, but if a site is unable to accommodate basic needs to set up a successful practice, consider moving on to identify other sites

